# Effects of an intervention combining physical activity and components of Amygdala and Insula Retraining (AIR) on sleep and working memory among older male adults

**DOI:** 10.3934/Neuroscience.2024025

**Published:** 2024-10-12

**Authors:** Monireh Asadi Ghaleni, Forouzan Fattahi Masrour, Narjes Saryar, Alexandra J. Bratty, Ebrahim Norouzi, Matheus Santos de Sousa Fernandes, Georgian Badicu

**Affiliations:** 1 Department of Motor Behavior, Ferdowsi University of Mashhad, Mashhad, Iran; 2 Department of Exercise Physiology, Central Tehran Branch, Islamic Azad University, Tehran, Iran; 3 Department of Sport Management, University of Eyvanakey, Tehran, Iran; 4 AB Research Consulting, New York, U.S; 5 Department of Physical Education, Farhangian University, Tehran, Iran; 6 Keizo Asami Institute, Federal University of Pernambuco, Recife, Pernambuco, Brazil; 7 Department of Physical Education and Special Motricity, Faculty of Physical Education and Mountain Sports, Transilvania University of Brasov, 500068 Brasov, Romania

**Keywords:** older, physical activity, amygdala and insula retraining, depression, emotion

## Abstract

**Background:**

Older individuals are at a particular risk of sleep disorders, a loss of cognitive and emotional control, and a poor quality of life. Pharmaceutical therapy for these conditions is commonplace but has not been particularly effective, and relatively little research exists for their treatment using non-pharmacological approaches. The effectiveness of Physical Activity plus selected components of Amygdala and Insula Retraining (PAAIR) was tested to improve sleep quality, depression, working memory, and emotion regulation among older males.

**Methods:**

This was a parallel, randomized control trial. The study was conducted in-person among 40 older Iranian men (*M*_age_: 65.78, *SD* = 2.41). The participants were randomly assigned with equal allocation to either the PAAIR or a control condition. Both interventions were conducted in-person over 12 weeks. The participants met twice weekly for 45-minute sessions at a local elderly training and rehabilitation center. All participants completed measurements for sleep quality, depressive symptoms, working memory, and emotion regulation at baseline, 12 weeks (immediately after the intervention), and 8 weeks later.

**Results:**

Among the 36 individuals who finished the study, their sleep quality, working memory, and emotion regulation improved, and their depressive symptoms were reduced from baseline to 12 weeks (post-intervention) and 8 weeks later; these effects were seen even more so for the PAAIR group compared to the control group, with large to extremely large effect sizes.

**Conclusion:**

The findings suggest that PAAIR has the potential to enhance sleep quality, cognitive function, and emotion regulation and reduce depressive symptoms among older men, thus contributing to their quality of life and mental health.

## 1. Introduction

Older adults frequently experience impaired cognition and a decreased quality of life. Studies indicate that sleep quality, cognitive function, and mental health are significantly reduced among older individuals [1],[2]. Older people have higher rates of dementia, cognitive decline, psychological distress, mood disturbances, and anxiety and depression symptoms [3]–[6]. Additionally, older individuals are less physically active than younger individuals [1], and research suggests that physical inactivity can lead to declining physical health, thus negatively impacting daily life and cognitive functioning [4].

Understanding the interconnected nature of health concerns among older individuals requires recognizing the complex web of challenges they often face. Sleep disorders, such as insomnia and sleep apnea, can disrupt restorative sleep [7], thus contributing to cognitive decline and memory issues associated with conditions such as Alzheimer's disease and dementia [8],[9]. Additionally, these cognitive challenges can lead to emotional instability, thereby exacerbating mental health concerns such as depression and anxiety [10],[11]. Moreover, sensory and visual impairments, and chronic conditions such as cardiovascular disease, diabetes, and arthritis, are prevalent among older individuals and can further impact physical mobility and emotional well-being [12].

Numerous approaches have been explored to address these interrelated conditions in older adults. Multimodal treatments, including medication, counseling, and occupational therapy, are frequently used to prevent emotion dysregulation and cognitive dysfunction [13]. Pharmacological treatments are common but have not been particularly successful [14]. Non-pharmacological approaches, such as psychotherapeutic and behavioral treatments, are widely suggested [1]. However, research into their potential as supplemental or alternative therapies is still ongoing [15],[16]. Given that older adults comprise 10% of the general population and often experience an impaired quality of life, examining more non-pharmacological interventions among this group is a worthwhile endeavor.

The present study focuses on an intervention that combines physical activity with a neuroplasticity protocol. Previous research suggests that exercise is a viable non-pharmacological strategy to prevent age-related neurodegenerative disorders and cognitive decline [17]. Preliminary evidence indicates that exercise may enhance sleep and improve cognitive function [18]. For example, older individuals with minor cognitive impairments showed significant improvements in sleep quality and cognitive function after a 20-week physical activity program compared to a control group [19]. The positive outcomes of physical activities on cognition and motor functioning have also been reported with more conventional training techniques (including aerobic exercise, stretching, and spine flexibility) [20]. Additionally, higher physical activity levels have been correlated with an improved emotional well-being [21]. Longitudinally, older people who transitioned from some activity to none showed more negative emotional changes, while those who maintained or adopted activity experienced better outcomes. Additionally, regular physical activity has improved emotion regulation and empathy in individuals with multiple sclerosis, thus indicating exercise's potential to address emotional and social challenges [22].

Regarding the neuroplasticity protocol, there is evidence that the Amygdala and Insula Retraining (AIR) program positively affects physical and mental health [23],[24],[26]. AIR is hypothesized to enhance neurological inhibitory processes in areas of the prefrontal cortex, insula, and anterior and posterior cingulate [25]. Amygdala and Insula Retraining (AIR), which is commercially known as The Gupta Program, is a neuroplasticity-based intervention designed to address chronic pain. AIR employs neural rewiring techniques and supportive strategies such as cognitive reframing, mindfulness meditation, and guided imagery to modulate the central nervous system's response to pain. While the precise mechanisms of AIR remain under investigation, the foundational hypothesis suggests that the intervention facilitates the formation of new neural pathways associated with pain regulation and potentially inhibits established pain pathways (Gupta, 2002, 2010; Gupta et al., in publication). This modulation may involve strengthening the prefrontal cortex's inhibitory control over the limbic system, including the amygdala and insula (Bratty, 2024; Gupta et al., in publication; Sehlmeyer et al., 2009). These brain regions play pivotal roles in pain processing and emotional regulation (Sanabria-Mazo et al., 2020). Gupta's theory offers a novel perspective, thereby suggesting that fibromyalgia may stem from maladaptive neural circuits in the brain, particularly those involving the amygdala and insula (Kioussis & Pachnis, 2009; Kraus et al., 2021), which are associated with pain processing and emotional regulation (Meulders, 2020; Zaman et al., 2015). AIR is a brain retraining intervention developed in accordance with this theory, and aims to recalibrate these neural circuits to reduce the brain's exaggerated responses to pain and stress (Steinman, 2004). In addition to standard medical care, experimental and longitudinal studies demonstrated that AIR appeared to have a positive impact on managing pain, fatigue, and depressive symptoms [24],[26]. Moreover, AIR improved the scores for physical health, pain, vitality, distressing symptoms, and exhaustion compared to standard care [27]. Other research has supported the theory of immune conditioning in the insula [28], which is the foundational premise of the AIR program.

Previous studies suggest that physical activity and AIR therapy can promote physical and mental health. The idea to combine these protocols in one intervention was based on outcomes from prior research that highlighted the difficulty of motivating exercise in older people due to physical limitations [12],[29], while AIR has successfully emphasized the benefit of coupling neuroplasticity processes with mild movements [23]–[26]. Thus, it was hypothesized that combining physical activity with a neuroplasticity protocol could provide a smoother transition to better quality living among older people.

In general, physical activity and neuroplasticity interventions are low-cost and widely accessible, thus benefiting older individuals, their families, communities, and public health. However, little is known regarding which pursuits are most advantageous for this population [1], or whether combining these interventions would improve sleep quality, cognitive function, and emotional stability. Consequently, the current study's goal is to examine the impact of physical activity plus the AIR intervention (PAAIR) on sleep quality, depressive symptoms, working memory, and emotion regulation among older adults. The following hypotheses are explored:

H1: There will be a significant improvement in participants' sleep quality, depressive symptoms, working memory, and emotion regulation after the PAAIR intervention.

H2: In comparison to a control group, those receiving the PAAIR intervention will experience a significant improvement in sleep quality, depressive symptoms, working memory, and emotion regulation.

## 2. Methods

### 2.1. Trial design and participants

The current research is a parallel, randomized controlled trial with an equal allocation ratio of participants to either an intervention or a control group. A total of *N* = 72 older men were approached by telephone and invited to participate in the present study. An initial screening was used to determine the study eligibility. The inclusion criteria included the following: 1) male; 2) 65 years or older; 3) mental state testing score of 24 or more as measured by the Mini-Mental State Examination (MMSE); 4) good physical condition as measured by the Persian version of the Baecke questionnaire [30]; and 5) good physical activity status, also measured by the Persian version of Baecke questionnaire [31]. The exclusion criteria included the following: 1) female; 2) less than 65 years old; 3) participating in another RCT; 4) Alzheimer's disease; and 5) having a disorder such as posttraumatic stress, severe depressive illness as determined by The Diagnostic and Statistical Manual of Mental Disorders, Fifth Edition (DSM-5), or substance abuse. Recruitment took place between February 2022 and May 2022. Only male participants were included because of cultural considerations. In Iran, men and women cannot exercise together. Therefore, the Physical Activity (PA) component of the intervention could not be conducted with a mixed gender group, and only men were recruited for the current study.

The sample size was calculated with G*power, assuming two groups, three assessments per group (baseline, 12 weeks, 8 weeks later [follow-up]), a power (beta) of 0.95, an alpha of 0.05, and an effect size of 0.25 (Gignac & Szodorai (2016)). These parameters indicated that the total minimum sample size required was *N* = 36 [32]. Twice as many people were initially recruited and screened for the study because physical activity interventions had a dropout rate of about 30% [33]. The participants were randomized to groups by drawing tickets with sequential numbers on them from a ballot box. The odd numbers were allocated to the PAAIR group, while the even numbers were allocated to the control group.

**Figure 1.**
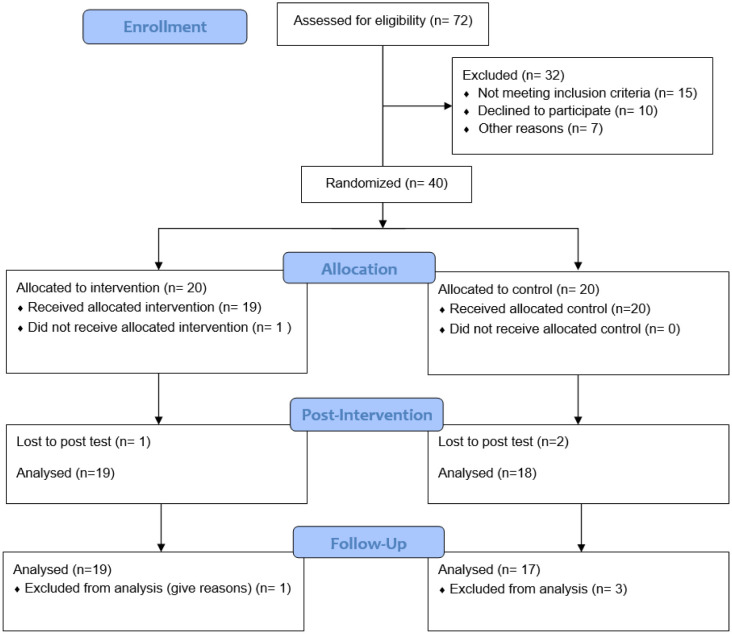
Flowchart of Participants.

Before signing a written consent form, the eligible participants were fully informed about the aims of the present research, as well as the anonymous methods used for data collection and handling. The Ferdowsi University of Mashhad's Review Board (Mashhad, Iran) approved the study, which was conducted in compliance with the ethical standards stated in the seventh and most recent edition of the Declaration of Helsinki (2013). The participants completed questionnaires which assessed their sleep quality, depressive symptoms, and emotion dysregulation. Additionally, they completed a computer-based working memory test. The measurements were conducted at baseline, after the intervention (12 weeks), and once more 8 weeks later. Figure 1 displays the flow of the participants in the study. Initially, *N* = 72 participants were recruited. However, *n* = 32 subjects were excluded for either not meeting the screening criteria, declining to participate, or some other reason. Therefore, a total of *N* = 40 men participated. Of these, 20 were randomly assigned to the PAAIR group and 20 were assigned to the control group. Upon the conclusion of the study, *n* =19 participants remained in the PAAIR group and *n* = 17 remained in the control group.

### 2.2. Outcome measures

#### 2.2.1. Sleep quality

The sleep quality was assessed using the Persian version of the Pittsburgh Sleep Quality Index (PSQI) [34]. It is a 7-item scale that assesses various dimensions of sleep: 1) subjective sleep quality; 2) sleep latency; 3) duration of sleep; 4) sleep efficiency; 5) sleep disturbances; 6) sleeping pills; and 7) poor daytime functioning. The participants rated each item on a 4-point Likert scale, with 0 being the most favorable (*good sleep quality*) to 3 being the least favorable (*poor sleep quality*). Ratings were added to create a global score between 0 and 21 for each participant. A score greater than 6 suggested poor sleep quality. Previous research indicated that the Persian PSQI had a good internal consistency (α = .83) [35]. In the present study, internal reliability scores were also acceptable at each point of measurement (baseline α = .82, 12 weeks α = .79, and 8-week follow-up α = .81).

#### 2.2.2. Depressive symptoms

The Persian version of the Beck Depression Inventory (BDI) [36], which has been validated for Iranian populations [37], was used to measure depressive symptoms. The following symptoms were evaluated using the 13-item scale: 1) sadness; 2) hopelessness; 3) diminished sexual function; 4) diminished motivation; 5) loss of appetite; 6) difficulties in attention and concentration; 7) lassitude; 8) inability to feel; 9) pessimistic thoughts; 10) suicidal thoughts; 11) diminished sleep; 12) diminished interest; and 13) feelings of guilt. The responses were recorded using a 4-point Likert-type scale from 0 (*not at all*) to 3 (*definitely*) and summed to create an overall score. The overall scores of 0–13 denoted no depression, 14–19 denoted mild depression, 20–28 denoted moderate depression, and 29–63 denoted severe depression. Previous research [37] demonstrated a good internal reliability for the scale (α = 0.89). In the present study, the internal reliability scores were also acceptable at each point of measurement (baseline α = .90, 12 weeks α = .87, and 8-week follow-up α = .84).

#### 2.2.3. Working memory

The N-back task [38] was used to measure the working-memory performance. The N-back task displayed letters and numbers in a specific order three times for the participants. Numbers/letters were presented to older adults, who were asked to determine if they matched the numbers/letters that were provided in one or two previous trials. In every trial, the participants responded by pressing a yes or no button. The participants finished the task for two levels of difficulty (one-back and two-back). Blue letters (vertical visual angle = 0.7°) on a light black background served as the stimuli. Each sequenced number/letter was shown for 1,000 milliseconds, with a 2,500 millisecond response window. The inter-stimulus interval was set to 2,000 milliseconds. A letter/number matching that previously provided N trials occurred at a rate of 25% in each block. The average reaction time for correct responses was used to calculate the scores. The test-retest reliability of the measure was confirmed in the present study (one-back, *r* =.78; two-back, *r* =.75).

#### 2.2.4. Emotion dysregulation

The Persian version of the difficulties in the emotion regulation scale (DERS) was used to assess emotion dysregulation [39]. It is a 36-item scale used to measure emotion dysregulation across six categories: 1) avoidance of negative emotions; 2) difficulties engaging in goal-directed behaviors; 3) difficulties controlling impulsive behaviors; 4) limited access to emotion regulation strategies; 5) lack of emotional awareness; and 6) low emotional clarity. Each question was answered by respondents using a 5-point Likert-type scale ranging from 1 (*almost never*) to 5 (*almost always*): the higher the score, the lower the emotion regulation [40]. Prior research indicated that the Persian version of DERS had an acceptable internal reliability (α = .93) [41]; in the current study, the internal reliability scores were acceptable at each point of measurement (baseline α = .91, 12 weeks α = .90, and 8-week follow-up α = .86).

### 2.3. Intervention

#### 2.3.1. Physical activity plus Amygdala & Insula Retraining (PAAIR) intervention

The PAAIR group received an aerobic activity plus selected components of the amygdala and insula retraining (AIR). The respondents participated in two sessions per week in person and met as a group at the Yadgar Elderly Training and Rehabilitation Center (Mashhad, Iran) for 3 months, where they engaged in a moderate-intensity aerobic physical activity for 30 minutes following approximately 15 minutes of education about the AIR program.

The physical activity focused on improving the overall functional fitness, aerobic capacity levels, balance, and flexibility, and consisted of four main parts: 1) a warm-up; 2) a static aerobic exercise (bodyweight exercises, mountain climber twist, plank, plank-to-knee tap, skaters, stretching); 3) a dynamic aerobic exercise (walking, running, and cycling); and 4) a cool down. The exercises were performed with the guidance of sports instructors and in accordance with each participant's physical fitness levels. Gradually increasing the intensity and complexity of exercises over time was encouraged depending on participants' capability.

The AIR program is comprised of specialized neuroplasticity techniques aimed at retraining the immune system and the nervous system's hyperactivity, and is supported by practices such as breathing, meditation, and neurolinguistic programming. The full AIR protocol has yielded positive outcomes in prior studies [42]. In the current study, selective parts of it were shared during the bi-weekly 15-minute education sessions. The participants learned about the following topics and were encouraged to engage in them further in their own time. The overall content and structure closely tracked with that used for AIR in a prior study [27]. In the first week, the theory of how the brain and the limbic system respond to fear and conditioning were discussed. In week 2, interrupting negative beliefs, thoughts, and emotions, and their bodily impact were discussed. Body scans and meditations were covered in week 3. Walking meditation and the anchoring presence in the body were considered in week 4. In week 5, self-regulation through mindfulness practices was discussed. In weeks 6 and 7, becoming aware of unpleasant thoughts triggered by external events and excessive nervous system reactions were trained. Additionally, the core 7-step brain retraining process was taught to the participants, who were then asked to use this fundamental neuroplasticity technique each day for the remainder of the intervention. In weeks 8 to 10, to the process to recognize and modify thoughts with the brain retraining process was reviewed, along with motivation, quality of life, values, mindfulness, acceptance, and meditation. In weeks 11 and 12, the method to develop a positive vision for the future and a review of the AIR practice were considered. AIR components such as recommendations for improving sleep and nutrition and spending time in nature were not incorporated in this study. In addition to the AIR information shared during the two sessions per week, the participants were asked to practice aspects of the AIR program 20–60 minutes daily in their own time. Two clinical psychologists familiar with its protocols administrated the AIR intervention.

#### 2.3.2. Control condition

The participants of the control condition met for approximately 45 minutes twice per week in person at the Yadgar Elderly Training and Rehabilitation Center (Mashhad, Iran). They engaged in group discussions about their health statuses and everyday lives. Moreover, they participated in inactive pursuits, such as meal-planning, listening to music, and keeping diaries. Additionally, they could talk with nurses and medical doctors about their health, their blood pressures were checked weekly, and counseling for concerns related to daily living was provided.

### 2.4. Analyses

A series of two-way mixed ANOVAs were performed for time (baseline, 12 weeks, 8-week follow-up) and group (PAAIR vs. Control) for each of the dependent variables: sleep quality, depressive symptoms, working memory, and emotion regulation. Shapiro-Wilk tests indicated that the data for each outcome variable were normal. Post-hoc analyses were conducted using independent and paired *t* test corrections for the interaction effects. Partial eta-squared coefficients were used to estimate the effect sizes, whereas Cohen's *d* values were used for pairwise comparisons. Each statistical analysis was conducted using IBM SPSS® 20.0, with a *p* ≤ 0.05 level of significance.

### 2.5. Sample characteristics

*N* = 72 older men were recruited, *N* = 40 of them qualified to participate in the study, with *n* = 20 allocated to each group. There was no significant difference between the two groups for age, marital status, education, or time since retirement (see Table 1). *N* = 36 participants completed the study (*n* = 19 PAAIR, *n* = 17 control). Therefore, subsequent analyses were based off this final participant number.

**Table 1.** Baseline sociodemographic of study participants.

**Dimension**	**Group**		**Statistics**
	**PAAIR**	**Control**	
N	20	20	
Age (years): *M (SD)*	66.21 (2.82)	65.13 (2.76)	
Age range (years)	65-73	65-72	
Time since retirement (years): *M (SD)*	5.13 (1.3)	4.93 (1.2)	
Marital status (single/married): *n*	5/15	4/16	
Education (middle school/high school/bachelor): *n*	4/8/8	5/9/6	*t*(38) = 1.66, *p* = .12, *d* = 0.41 *t*(38) = 0.71, *p* = .48, *d* = 0.27 χ2(1) = 0.51, *p* = 0.24 χ2(2) = 0.46, *p* = 0.80

*Note*: PAAIR = Physical activity plus Amygdala Insula Retraining

## 3. Results

### 3.1. Sleep quality

Regardless of the group, the sleep quality scores significantly decreased (i.e., sleep improved) across time with an extremely large effect size (*F*(2, 76) = 54.46, *p* < .001, partial η^2^ = .63). The impact of time was entirely attributed to fewer sleep problems in the PAAIR group with a very large effect size (*F*(1, 38) = 12.64, *p* = .001, partial η^2^ = .48). Moreover, there were significant interaction effects with an extremely large effect size (*F*(2, 76) = 36.17, *p* < .001, partial η^2^ = .51). Post hoc calculations (paired *t* tests) indicated that the sleep quality scores significantly decreased in the PAAIR group from the beginning until after the intervention with a large effect size (*t*(18) = 7.63, *p* < .001, *d* = .98), and from the beginning to the follow-up point (8 weeks post-intervention) with a very large effect size (*t*(18) = 9.82, *p* < .001, *d* = 1.83). For the control group, the sleep quality significantly decreased from before to after the intervention with a small effect size (*t*(18) = 2.88, *p* < .001, *d* = .17), and approached significance from before the intervention to the follow-up point (*t*(17) = 1.89, *p* = .07, *d* = .17). Post-hoc (independent *t* tests) calculations which compared the PAAIR and control groups demonstrated no difference in the sleep quality at baseline (*t*(37) = 0.66, *p* = .50, *d* = .22), but the groups approached a significant difference at the follow-up point with a large effect size (*t*(37) = 3.44, *p* = .07, *d* = 1.09) and were significantly different at the follow-up point with a very large effect size (*t*(37) = 6.23, *p* < .001, *d* = 1.97), with the PAIIR group showing more improvements than the control group.

### 3.2. Depressive symptoms

The depressive symptoms declined with an extremely large effect size (*F*(2, 76) = 92.89, *p* < .001, partial η^2^ = .65) across time and regardless of the group. The impact of time was attributed to reduced symptoms among PAAIR participants (*F*(1, 38) = 4.31, *p* = .04, partial η^2^ = .24). Furthermore, the Time × Group interaction effect was significant with an extremely large effect size (*F*(2, 76) = 45.79, *p* < .001, partial η^2^ = .54). Post-hoc calculations (paired *t* tests) demonstrated that depression significantly dropped in the PAAIR group from before to after the intervention with an extremely large effect size (*t*(18) = 19.46, *p* < .001, *d* = 2.65), and from before the intervention to the follow-up point with a very large effect size (*t*(18) = 8.29, *p* < .001, *d* = 1.70). Moreover, the depressive symptoms significantly decreased in the control group from before to after the intervention with a small effect size (*t*(17) = 3.24, *p* = .004, *d* = .41), and from before the intervention to the follow-up point with a medium effect size (*t*(17) = 6.52, *p* < .001, *d* = .63). Post-hoc calculations (independent *t* tests) which compared the PAAIR and control conditions indicated that the depression scores were not different at baseline (*t*(37) = 1.71, *p* = .09, *d* = .54); however, they were significantly different between groups with very large effect sizes at post-intervention (*t*(37) = 6.16, *p* < .001, *d* = 1.95) and at follow-up (*t*(37) = 2.05, *p* = .04, *d* = 1.65), with the PAAIR group showing more improvements than the control group.

### 3.3. Working memory

The working memory significantly increased with a large effect size (*F*(2, 76) = 17.95, *p* < .001, partial η^2^ = .33) across time and regardless of the group. Again, the significant time effect reflected an improvement in the working memory in the PAAIR group with an extremely large effect size (*F*(1, 38) = 43.47, *p* < .001, partial η^2^ = .58). Moreover, the Time x Group effects were significant with a very large effect size (*F*(2, 76) = 19.91, *p* < .001, partial η^2^ = .44). Post-hoc calculations (paired *t* tests) showed that the working memory performance significantly increased within the PAAIR group with very large effect sizes from before to after the intervention (*t*(18) = 6.11, *p* < .001, *d* = 1.71), and from before the intervention to the follow-up point (*t*(18) = 6.61, p < .001, *d* = 1.64). The working memory performance in the control group was non-significant from baseline to post-intervention (*t*(17) = 0.72, *p* = .47, *d* = .13), and from before the intervention to the follow-up point (*t*(17) = 0.64, *p* = .52, *d* = .17). Post-hoc calculations (independent *t* tests) between the PAAIR and control conditions suggested that there was no significant difference in the working memory before the intervention (*t*(37) = 1.33, *p* = .19, *d* = .43); however, there were significant differences between groups with extremely large effect sizes at post-intervention (*t*(37) = 6.82, *p* < .001, *d* = 2.23) and at follow-up (*t*(37) = 7.31, *p* < .001, *d* = 2.36), with the PAAIR group demonstrating more improvements than the control group.

### 3.4. Emotion regulation

Emotion dysregulation significantly decreased with an extremely large effect size (*F*(2, 76) = 116.09, *p* < .001, partial η^2^ = .58) across time and regardless of the group. There was a significant reduction in emotion dysregulation in the PAAIR group with an extremely large effect size (*F*(1, 38) = 75.13, *p* < .001, partial η^2^ = .54). Moreover, there was a significant interaction effect of Time and Group with a very large effect size (*F*(2, 76) = 114.10, *p* < .001, partial η^2^ = .49). Post-hoc calculations (paired *t* tests) indicated that emotion dysregulation was significantly reduced in the PAAIR group with extremely large effect sizes from before to after the intervention (*t*(18) = 13.23, *p* < .001, *d* = 2.77), and from before the intervention to the follow-up point (*t*(18) = 13.73, *p* < .001, *d* = 2.99). In the control group, the emotion dysregulation scores were nonsignificant from before to after the intervention (*t*(17) = 0.25, *p* = .79, *d* = .03), and from before the intervention to the follow-up point (*t*(17) = 0.04, *p* = .96, *d* = .05). Post-hoc calculations (independent *t* tests) between the PAAIR and control conditions demonstrated that emotion dysregulation scores were not different at baseline (*t*(37) = 1.30, *p* = .20, *d* = .41); however, they were significantly different with extremely large effect sizes at the post-intervention (*t*(37) = 10.54, *p* < .001, *d* = 3.13) and follow-up measurements (*t*(37) = 12.02, *p* < .001, *d* = 3.22), with the PAAIR group demonstrating more improvements than the control group.

The mean scores for all the outcome variables at all-time points are represented in Table 2. The absolute reduction in the mean scores for all measures from before to after the intervention was between six to 85 times greater for the PAAIR group than the control group. From baseline to follow-up the absolute reduction in mean scores ranged from three to 54 times greater for the PAAIR group than the control group.

**Table 2.** Means and standard deviations for sleep quality, depressive symptoms, working memory and emotion dysregulation.

	Groups	

	PAAIR (*n* = 19) *M (SD)*	Control (*n* = 17) *M (SD)*
Sleep quality		
Baseline	13.75 (4.75)	14.70 (4.20)
Post-intervention	9.20 (4.49)	13.95 (4.22)
Follow-up	6.10 (3.50)	13.95 (4.40)
Total Decrease	-7.65	-0.75
Percent Decrease	56%	5%
Depressive symptoms		
Baseline	18.90 (4.41)	16.80 (3.23)
Post-intervention	9.40 (2.47)	15.40 (3.57)
Follow-up	12.35 (3.18)	14.60 (3.71)
Total Decrease	-6.55	-2.20
Percent Decrease	35%	13%
Working memory		
Baseline	2.34 (0.36)	2.48 (0.27)
Post-intervention	1.80 (0.26)	2.44 (0.31)
Follow-up	1.73 (0.38)	2.53 (0.29)
Total Decrease	-0.61	0.05
Percent Decrease	26%	-2%
Emotion dysregulation		
Baseline	131.25 (16.74)	137.80 (15.01)
Post-intervention	84.45 (17.00)	137.25 (14.55)
Follow-up	82.65 (13.89)	136.90 (15.13)
Total Decrease	-48.6	-0.90
Percent Decrease	37%	1%

*Note*: PAAIR = Physical activity plus Amygdala Insula Retraining. Total Decline = difference between Baseline and Follow-up. Percent Decrease = percentage change between Baseline and Follow-up.

## 4. Discussion

The present study's goal was to evaluate the impact of the PAAIR intervention on sleep quality, depressive symptoms, working memory, and emotion regulation compared to a control group among older men. The results supported both hypotheses. First, there was a significant improvement for all outcome variables following the PAAIR intervention. Second, in comparison to the control group, the PAAIR intervention significantly improved all outcome variables with large to extremely large effect sizes. Notably, these improvements held for 8 weeks after the intervention.

The current results contribute to the literature by demonstrating that PAAIR can be an effective intervention for older men. Such results are relevant because the older population is at increased risk for loss of cognitive and emotional control, depression, and sleep disturbances. The present study is in line with previous research which claimed that physical activity should be combined with lifestyle and cognitive emotion changes to be more effective [43]. Indeed, the current research offers a possible method to improve the physical and mental health of the older population, which may facilitate greater longevity, independence, and productivity.

PAAIR's effectiveness in improving sleep quality and diminishing depressive symptoms could be explained by supporting research. For example, physical activity has been associated with reduced depressive symptoms [44] and reduced sleep problems [45]. Moreover, AIR has demonstrated significant reductions in depression [23] and fatigue [24],[26] compared with control groups. Additionally, it is hypothesized that both amygdala and insula retraining and physical activity can reduce the overall levels of inflammation, which is a crucial factor in reducing depression and sleep problems [43]. Therefore, PAAIR can potentially be an effective resource for brain health by reducing inflammation [27],[46],[47].

Furthermore, the effectiveness of PAAIR on sleep quality may be due to its impact on rumination and dysfunctional thoughts, which are considered the main reasons for sleep disorders [48]. Other research has demonstrated that physical activity and components of AIR, such as mindfulness, can improve sleep and reduce rumination [49] and depression [46]. Indeed, mindfulness training encourages individuals to accept their emotions and feelings. This acceptance enables an increased psychological flexibility and improves the sleep quality by reducing destructive sleep-related thoughts.

Regarding PAAIR's impact on cognitive function, neurophysiology studies have demonstrated that physical activity can enhance blood flow in the brain and levels of norepinephrine and dopamine [50],[51]. Moreover, neuroplasticity training, such as AIR, can change the brain structure, particularly in the prefrontal cortex, which plays a key role in cognition [17],[28],[52]. Some of these structural changes contribute to improvements in the working memory [51]. Additionally, diabetes, cardiovascular disease, and high blood pressure are expected to harm global cognitive functions in older people [51]. A primary purpose of physical activity is to prevent or mitigate these diseases and their adverse effects on cognitive functioning [50]. Thus, by combining exercise and neuroplasticity training, the PAAIR intervention can improve the working memory.

Finally, emotional control may have improved from using PAAIR because some of AIR's neuroplasticity techniques, such as acceptance and mindfulness, have demonstrated positive changes in self-regulation and the processing of emotions [53]. Additionally, setting goals and values during mindfulness training can facilitate changes in behavior, whereby previously sedentary individuals are motivated to be more active and regularly engage in exercise [54]. Thus, some AIR components can facilitate emotion regulation and help older people initiate and maintain their physical activity, thereby allowing individuals to benefit from the combined physical activity and AIR intervention.

Our research aligns with Gupta's theory (2002, 2010) by demonstrating that AIR significantly alleviates sleep and cognitive problems beyond what is typically achieved solely with standard medication. By targeting the dysfunctional neural pathways described in Gupta's theory, AIR offers a novel approach to modify the brain's response to chronic pain and emotional distress (Bennett, 2019; Mobbs et al., 2015). Physical activity further complements this approach by promoting neuroplastic changes, increasing pain tolerance, and improving the overall function (Hötting & Röder, 2013). The combined application of these interventions seems to more comprehensively address both the neurological and physiological aspects of aging, potentially shifting away from traditional pharmacological treatments. Future research should continue to explore these approaches, thereby focusing on how they can be optimized and integrated based on Gupta's theoretical framework to enhance the patient outcomes in various populations.

The encouraging findings from this study should be considered in conjunction with its limitations. First, only self-reported measures were used instead of either collecting health professionals' ratings for the participants' symptoms or measuring objective biomarkers, such as cortisol and the brain-derived neurotropic factor (BDNF). Second, there was no way to evaluate the separate and relative impacts of AIR and physical activity on outcomes because these programs were combined in the tested intervention. Third, we only assessed older males; therefore, these results are not generalizable, and it is unknown if similar results would be observed among older females. Fourth, it is yet undetermined if the present results can be reproduced in other populations, such as people with mental illness or those with mild cognitive declines. Consequently, future research should endeavor to remedy the shortcomings of this study, including single- or double-blinded procedures and separate evaluations of AIR versus physical activity on the outcome variables.

## 5. Conclusion

Among older men, PAAIR significantly and positively impacted the sleep quality, depressive symptoms, working memory, and emotion regulation. Moreover, the PAAIR intervention significantly improved all measures compared to a control group, with large to extremely large effect sizes. Importantly, the positive impact of PAAIR was still observed 8 weeks following the intervention. These results are pertinent because older adults are more prone to mental health and cognitive issues and sleep disorders. PAAIR offers a viable means to improve these factors for older people. However, further investigations are necessary to evaluate the impact of PAAIR and other interventions, such as behavioral, cognitive, emotional, and social interventions, to support the older population as they age.

## Author Contributions

Conceptualization, M.A.G. and F.F.M; methodology, E.N.; software, E.N.; validation, N.S, G.B. and M.A.G.; formal analysis, F.F.M; investigation, N.S.; A.J.B, E.N; data curation, E.N.; writing—original draft preparation, E.N., A.J.B, G.B., M.A.G; writing—review and editing, G.B., M.S.S.F., A.J.B, F.F.M, visualization, N.S., E.N., M.S.S.F., M.A.G.; supervision, G.B. and E.N.; project administration, M.A.G. All authors read and approved the final manuscript.

## Data Availability

The datasets used and/or analyzed during the current study available from the corresponding author on reasonable request.

## Institutional Review Board Statement

The Review Board of the Ferdowsi University of Mashhad, (Mashhad, Iran) approved the study, which was performed in accordance with the ethical principles laid down in the seventh and current edition (2013) of the Declaration of Helsinki (protocol code: IR1400.12231.538. date of approval: 14.01.2022). Informed consent was obtained from all subjects involved in the study.
